# Psychological Status Among Anesthesiologists and Operating Room Nurses During the Outbreak Period of COVID-19 in Wuhan, China

**DOI:** 10.3389/fpsyt.2020.574143

**Published:** 2020-12-03

**Authors:** Xin-yi Li, Jin Wang, Rui-xian Zhang, Luhua Chen, Colin K. He, Cheng-yao Wang, Jian-juan Ke, Yan-lin Wang, Zong-ze Zhang, Xue-min Song

**Affiliations:** ^1^Department of Anesthesiology, Zhongnan Hospital of Wuhan University, Wuhan, China; ^2^Yunnan Center for Disease Control and Prevention, Kunming, China; ^3^Department of Psychology, Faculty of Social Sciences, State Key Laboratory of Brain and Cognitive Sciences, The University of Hong Kong, Pokfulam, Hong Kong; ^4^Department of Statistics & Machine Learning, Stego Tech LLC, King of Prussia, PA, United States

**Keywords:** COVID-19, anesthesiologists, depression, anxiety, social support

## Abstract

**Background:** Coronavirus Disease 2019 (COVID-19) caused by a novel strain of coronavirus (SARS-CoV-2) posed a major threat to public health. Anesthesiologists and operating room (OR) nurses are at high risk of occupational exposure to SARS-CoV-2 and developing COVID-19. We conducted a single-center survey to investigate the psychological status and perceived social support among operation room (OR) medical staffs during the outbreak of Coronavirus Disease 2019 (COVID-19).

**Methods:** A total of 197 OR medical staffs were enrolled in the survey. The authors performed a cohort study during the period of Wuhan lockdown and then conducted a longitudinal follow-up after lifting of lockdown. The Patient Health Questionaire-9 (PHQ-9) was used to assess for depression and Generalized Anxiety Disorder-7 (GAD-7) for anxiety. The Multidimensional Scale of Perceived Social Support (MSPSS) was used to assess perceived social support. We compared the psychological status of OR medical staffs before and after lifting of Wuhan lockdown.

**Results:** During the period of city lockdown, 177 (89.8%) had close contact with confirmed COVID-19 cases. The prevalence of depression and anxiety in OR medical staffs was 41.6 and 43.1% under Wuhan lockdown, while 13.2 and 15.7% after lifting of lockdown (*P* = 0.002, *P* = 0.004). Logistic regression analysis showed that being female, living in suburb areas, shortage of protective equipment and close contact with COVID-19 patients were associated with a higher risk of depression and anxiety. Perceived social support was negatively correlated with depression and anxiety severity in the OR medical staffs (*P* < 0.05).

**Conclusions:** OR medical staffs exhibited high incidence of anxiety and depression faced with the high risk of exposure to COVID-19 patients. More social support and social recognition for anesthesiologists and OR nurses might potentially help them relieve their psychological pressure.

## Introduction

The outbreak of COVID-19 in the central Chinese city of Wuhan since December 2019 has been prompted worldwide concern, as it is an emergency threatening global public health (1–6). The Chinese government has adopted a series of measures to control this epidemic, including locking down the whole city of Wuhan. This 76-days lockdown imposed on the city has greatly affected the daily lives of the citizens, but finally brought a much-longed milestone in China's fight against the virus.

During the recent outbreak of COVID-19, anesthesiologists and OR nurses had perfomed anesthesia and surgical procedures for hundreds of COVID-19 patients in Wuhan. Besides, anesthesiologists also joined in the medical corps in Leishenshan and Fangcang hospital and provided care for COVID-19 patients as experts in emergent airway management and intensive care, which imposed a significant risk to their own health. Anesthesiologists and operating room (OR) nurses are exposed to droplet or blood-borne transmission of pathogens during endotracheal intubation and open surgical procedures, which put them at high potential of nosocomial infection (7–13). The operating room staffs thus face the risk of occupational exposure to COVID-19 patients.

Anesthetists often manage high-risk patients and emergency circumstances which makes them susceptible to stress and psychological problems (14–17). However, whether the COVID-19 epidemic could cause psychological problems among anesthesiologists and OR nurses is unknown. Besides, several studies have shown that perceived emotional and instrumental support had significantly protective effects on depression in medical staffs (18). In the later stage of the pandemic, medical staffs have gained more social support including a series of encouragements from the media and the whole society, more medical team reinforcements and the praise from the Chinese health authorities. In this study, we investigated the psychological status among the OR medical staffs in the context of COVID-19 outbreak and city lockdown. Furthermore, we assessed the effects of perceived social support on psychological status of the OR medical staffs.

## Methods

### Design, Participants, and Procedure

A pilot investigation was carried out in the Department of operating room, Zhongnan Hospital of Wuhan University, which included both anesthesiologists and operating room nurses. The study was approved by the Ethics Committee of Zhongnan Hospital of Wuhan University (No.2020029). The online questionnaire was given to the participants in the form of sojump. Sojump is an software to design the online questionnaires in Chinese (https://www.wjx.cn/). We conducted two survey using the same online questionnaires at two different time point. The first survey was performed at 7 weeks after lockdown of Wuhan city, and the questionnaire results were collected from March 12, 2020 to March 15, 2020. We retrieved a total of 204 questionnaires, seven incomplete questionnaires were eliminated. The remaining 197 questionnaires were completed eligibly, giving a response rate of 96.5%. This survey period corresponded to the reducing stage after the maximum point of the COVID-19 epidemic outbreak in China. One and a half months after the lifting of Wuhan lockdown (April 8, 2020), we conducted the secondary survey using the same online questionnaire, The results were collected from May 23, 2020 to May 26, 2020 as a longitudinal follow-up. The same participants enrolled in the first survey were invited to the secondary survey and fully completed. This survey period corresponded to the recovering stage after control of COVID-19 epidemic in Wuhan. The exclusion criteria were as follows: (1) participants who had not finished the questionnaire; (2) participants who were unable to finish the self-report questionnaire on their own due to a serious physical condition; (3) OR staff who were not at work.

### Questionnaire

The demographic questionnaire included gender, age, marital status, occupation, educational background, and underlying chronic disease (specifically, coronary artery disease, hypertension, or diabetes).

Patient Health Questionaire-9 (PHQ-9) was used to measure depression (19, 20). The questionnaire includes nine items and each is assessed on a 0 (lack of depression symptoms) to 3 (severe depression symptoms). Total score of the questionnaire ranges from 0 to 27. Generally, a score of 0–4 indicates no depression, 5–9 mild depression, 10–14 moderate depression, and 15–27 severe depression. According to PHQ-9, depression is a serious mental disorders typically marked by lack of self-confidence, dismay, sadness and grief.

Generalized Anxiety Disorder-7 (GAD-7) was used to assess the symptoms of anxiety (21). It contains seven items, and each question assessed on a scale from 0 (lack of anxiety symptoms) to 3 (severe anxiety symptoms). Generally, a score of 0–4 indicates no anxiety, 5–9 mild anxiety, 10–14 moderate anxiety, and 15–21 severe anxiety. According to GAD-7, anxiety is generalized as the onset of panic status and the manifestations of tension and inexplicable fear. The previous study has shown that this questionnaire can assess depression in Chinese general hospitals with good reliability and validity (19).

Multidimensional Scale of Perceived Social Support (MSPSS) was used to measure perceived support from family, friends and significant others (21). It is a self-report which are rated on a 7-point Likert scale, ranging from 1 (strongly disagree) to 7 (“strongly agree”). “Significant other” is intentionally undefined so that the respondent can identify their own significant other(s). The MSPSS is scored by summing the responses of the 12 items. Scores range from 12 to 84, and higher scores indicate higher levels of perceived social support.

### Data Analysis

SPSS 24.0 software (IBM, Armonk, NY) was used for statistical analysis. Data are presented as frequency and percentage with a 95% confidence interval or median and interquartile range for skewed continuous variables. Chi-square test was conducted to examine whether there were statistically differences in the prevalence of depression and anxiety between different group, or between the different time points.

Multivariable logistic regression was conducted to adjust for confounding of the observed baseline factors, and examine which factors were associated with each of the two outcomes of depression and anxiety (two separate models). Variables included in the regression model were defined a priori based on literature review and background knowledge. To evaluate the adjusted contribution of variables, we prespecified the variables that we postulated would be the strongly associated with depression and anxiety based on literature review and local knowledge: age, gender, education level and employment. These variables were included in the regression model regardless of the statistical significance, and other factors were put into the model using the forward selection method with a criterion of *P*-value <0.05. To validate the variables selection, the procedure was repeated in 1,000 bootstrapping.

A Mann-Whitney *U* test was used to compare group values for the PHQ-9, GAD-7 and MSPSS scores. Spearman correlational analysis was conducted to examine the correlations of perceived social support and psychological distress. A *P* < 0.05 was regarded as statistical significance.

## Results

### Participant Characteristics

A total of 197 OR medical staffs were evaluated in the study, including 61 (31.0%) anesthesiologists and 136 (69.0%) nurses. OR medical staffs in this cohort pertained only to anesthesiologists and OR nurses, not including surgeons. The demographics and clinical characteristics of the enrolled subjects are summarized in Table 1. The age of the enrolled subjects ranged from 21 to 58 years old. In the first survey, 158 (80.8%) worried about shortage of protection medical equipment, while 45 (22.8%) in the secondary survey (*P* < 0.001). In the first survey, 182 (92.4%) of participants considered that daily work was greatly impacted, while 68 (34.5%) in the secondary surve (*P* < 0.001). In the first survey, 122 (61.9%) staffs had close contact with confirmed COVID-19 cases, 8 (4.1%) were infected with SARS-CoV-2, including 7 (3.6%) anesthesiologists and 1 (0.5%) nurse. In the secondary survey, the number (177) and proportion (89.8%) of staffs who had close contact with confirmed COVID-19 cases increased, while the number of infected medical staffs remained the same as in the first survey. (We defined “close contact” as follows: 1. anesthesiologists and anesthesia nurses who performed endotracheal intubation for COVID-19 patients. 2. Surgery nurses who assisted surgeons in the surgical procedure for COVID−19 patients. We defined “without close contact” as follows: 1. Some of anesthesiologists, anesthesia nurses and surgery nurses who haven't participated in the operation for COVID-19 patients. 2. Instrument nurses without direct contact with COVID-19 patients, who were only involved in instruments preparation, drugs preparation and paper work).

**Table 1:** Baseline characteristics of 197 enrolled participants in the study.

**Characteristics *n* (%)**	**Under the lockdown**	**After lifting the lockdown**	***P*-value χ^2^ test**
**Gender**			
Male	48 (24.4)	48 (24.4)	
Female	149 (75.6)	149 (75.6)	
**Age (years)**			
20–40	167 (84.8)	167 (84.8)	
40–60	30 (15.2)	30 (15.2)	
**Education level**			
High school	3 (1.5)	3 (1.5)	
College	133 (67.5)	133 (67.5)	
Master	31 (15.7)	31 (15.7)	
PhD	30 (15.3)	30 (15.3)	
**Employment**			
Anesthetist	61 (31.0)	61 (31.0)	
Nurse	136 (69.0)	136 (69.0)	
**Marital status**			
Married	105 (53.3)	105 (53.3)	
Single	91 (46.2)	91 (46.2)	
Divorced	1 (0.5)	1 (0.5)	
**Living areas**			
Central area	115 (58.4)	115 (58.4)	
Suburb area	82 (41.6)	82 (41.6)	
**Having organic diseases**			
Yes	21 (10.7)	21 (10.7)	–
No	176 (89.3)	176 (89.3)	
**Infected with SARS-CoV-2**			
Yes	8 (4.1)	8 (4.1)	
No	189 (95.9)	189 (95.9)	
Close contact with COVID-19 cases			0.03
Yes	122 (61.9)	177 (89.8)	
No	75 (38.1)	20 (10.2)	
Shortage of protective equipment in work			<0.001
Yes	158 (80.2)	45 (22.8)	
No	39 (19.8)	152 (77.2)	
Depression symptoms			0.002
Yes	82 (41.6)	26 (13.2)	
No	115 (58.4)	171 (86.8)	
Anxiety symptoms			0.004
Yes	85 (43.1)	31 (15.7)	
No	112 (56.9)	166 (84.3)	
Daily work is greatly impacted			<0.001
Yes	182 (92.4)	68 (34.5)	
No	15 (7.6)	129 (65.5)	

### Prevelance of Depression and Anxiety Among OR Medical Staffs

**Figure 1:**
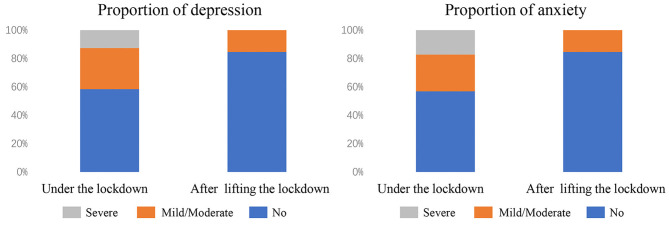
Proportion of depression and anxiety among OR medical staffs.

Among the enrolled 197 participants, 82 (41.6%) and 85 (43.1%) exhibited depression and anxiety under lockdown, while 26 (13.2%) and 31 (15.7%) exhibited depression and anxiety after lifting of lockdown (Table 1, Figure 1). Compared with their scores in the secondary survey, our study also observed that OR medical staffs in the first survey showed high scores in PHQ-9 items (*P* = 0.007) and GAD-7 items (*P* = 0.004), suggesting that OR medical staffs exhibited a higher degree of depression and anxiety during the COVID-19 epidemic and city lockdown, while their psychological distress were ameliorated after good control of epidemic and lifting the lockdown (Table 2).

**Table 2:** Psychological manifestations of OR medical staffs.

	**PHQ-9**	**GAD-7**	**MSPSS**
**Different time points**			
Under the city lockdown	7.5 (4.3–12.8)	8.7 (4.9–13.7)	53 (45–69)
After lifting the lockdown	4.2 (2.1–7.2)	4.3 (2.4–7.5)	72 (59–78)
*P*-value	0.007	0.004	0.011
**Different subgroups under the city lockdown**			
With close contact with COVID-19 cases	9.9 (6.2–14.4)	10.3 (7.1–14.9)	51 (42–65)
With no contact contact	5.5 (2.9–8.6)	5.8 (3.1–8.8)	55 (45–69)
*P*-value	0.005	0.006	0.232

*M (Q1–Q3): median and interquartile range*.

To further investigate whether close contact with COVID-19 patients affected the psychological status in the OR medical staffs, we divided the participants in the first survey into two subgroups: medical staff members who had close contact with COVID-19 patients and medical staff members without such close contact. There were also significant differences in PHQ-9 and GAD-7 scores between the two groups, suggesting that participants who had close contact with COVID-19 patients exhibited more depressive/anxious than those without close contact (*P* = 0.005, *P* = 0.006, Table 2).

### Perceived Social Support of OR Medical Staffs

Regarding perceived Social Support, OR medical staffs in the secondary survey exhibited higher MSPSS scores than those in the first survey (*P* = 0.011, Table 2). Howevere, there was no significant difference in MSPSS scores between the subgroups (with/without close contact with COVID-19 patiens).

### Factors Associated With Depression and Anxiety

All the data in the first survey were analyzed by multivariable logistic regression analysis. The results showed that being female, living in suburb areas, shortage of protective medical equipment and close contact with COVID-19 patients were associated with depression and anxiety. Women were susceptible to exhibit depression (OR, 1.62; 95% CI, 1.12–2.12; *P* = 0.035) and anxiety (OR, 1.73; 95% CI, 1.16–2.23; *P* = 0.041). Living in suburb areas was associated with more severe symptoms of depression (OR, 1.38; 95% CI, 1.03–1.72; *P* = 0.039) and anxiety (OR, 1.55; 95% CI, 1.28–1.84; *P* = 0.034). Shortage of protective medical equipment appeared to be a risk factor for all psychiatric symptoms after adjustment (OR, 1.88; 95% CI, 1.29–2.68; *P* = 0.023) and anxiety (OR, 2.08; 95% CI, 1.48–2.79; *P* = 0.017). Medical staffs who had close contact with COVID-19 patients were more prone to depression (OR, 2.52; 95% CI, 1.81–3.39; *P* = 0.005) and anxiety (OR, 2.67; 95% CI, 1.92–3.62; *P* = 0.002) than those without close contact (Table 3).

**Table 3:** Risk factors for psychological distress in logistic regression model.

**Variables**	**^a^Adjusted OR (95% CI)**	***P*-value**
**PHQ-9, depression symptoms**		
Gender (female vs. male)	1.62 (1.12–2.12)	0.035
Living areas (suburb vs. central area)	1.38 (1.03–1.72)	0.049
Shortage of protective equipment (yes vs. no)	1.88 (1.29–2.68)	0.023
Close contact with COVID-19 patients (yes vs. no)	2.52 (1.81–3.39)	0.005
**GAD-7, anxiety symptoms**		
Gender (female vs. male)	1.73 (1.16–2.23)	0.041
Living areas (suburb vs. central area)	1.55 (1.28–1.84)	0.034
Shortage of protective equipment (yes vs. no)	2.08 (1.48–2.79)	0.017
Close contact with COVID-19 patients (yes vs. no)	2.67(1.92–3.62)	0.002

a *Adjusted for gender, age, educational level, employment, marriage status, living areas, having organic diseases, when appropriate. 95% CI: 95% confidence interval*.

### The Correlation Analysis Between Psychological Distress and Perceived Social Support

There was an observed correlation between psychological distress, quality of life, and perceived social support using scales of PHQ-9, GAD-7 and MSPSS. Depression and anxiety were negatively correlated with perceived social support in both the participants who had close contact with confirmed COVID-19 cases and the participants without such close patients contact (Table 4).

**Table 4:** Correlation between perceived social support and psychological distress.

		**Depression**		**Anxiety**	
		***r* value**	***P*-value**	***r* value**	***P*-value**
OR medical staffs who had close contact with COVID-19 patients	Family support	−0.213	<0.001	−0.269	<0.001
	Friend support	−0.341	0.027	−0.353	<0.001
	Significant other support	−0.332	<0.001	−0.328	0.008
OR medical staffs who had no contact with COVID-19 patients	Family support	−0.227	<0.001	−0.272	<0.001
	Friend support	−0.276	<0.001	−0.244	<0.001
	Significant other support	−0.248	<0.001	−0.323	0.034

## Discussion

The main goal of this study is to investigate the psychological status and associated factors among operation room (OR) medical staffs during the outbreak of Coronavirus Disease 2019 (COVID-19). Facing the critical situations, being female, living in suburb areas, shortage of protective medical equipment and close contact with COVID-19 patients were associated with depression and anxiety of anesthesiologists and OR nurses. Anesthesiologists and OR nurses are at high risk of occupational exposure to infectious diseases (7–10). During the initial phase of the COVID-19 outbreak, the majority of hospitals in Wuhan were faced with a severe shortage of required personal protective equipment and SARS-CoV-2 Nucleic Acid Diagnostic Kits. This might explain why the eight medical staffs in our study have got infected without standard tertiary protection (PPE+N95mask+PAPR), after performing anesthesia for patients, who were confirmed by Nucleic Acid Diagnostic Kit several days later. In January, OR medical staffs in our hospital were directly caring for suspected or confirmed COVID-19 patients without standard tertiary protection, which might have put them at high risk of coronavirus infection during the operation. This specific situation caused considerable stress in our sample of OR medical staffs.

A series of studies have reported that potential exposure to infectious disease contributed to a certain degree of stress, uncertainty and psychological distress among the medical staffs (22–24). Lee et al. reported that health care workers who performed MERS-related tasks experienced depression and anxiety during the period of the MERS outbreak (22). Chen et al. have reported that nursing staffs experienced depression and anxiety during the period of the SARS outbreak. These previously published data have suggested that a proportion of medical staffs are prone to suffer from psychological disorders under the threat of the fatal virus infection (23). In our study, the prevelance of depression and anxiety among OR medical staffs were 41.6 and 43.1% under the city lockdown. As the better control of the COVID-19 epidemic, the prevalence of depression and anxiety decreased after lifting the lockdown. Although our observational data cannot prove a causal relationship, it is likely that he pandemic of COVID-19 has burdened an unprecedented psychological stress on OR medical staff, even produced a long-term post-traumatic stress disorder. Our results are consistent with the previous reports during the SARS and MERS outbreak (22–24).

Moreover, our study observed that being female, living in suburb areas, shortage of protective medical equipment and close contact with COVID-19 patients were associated with depression and anxiety. Zhang et al. reported that female health workers were 1.8 times as likely to suffer anxiety and depression than male health workers during the epidemic of COVID-19 (25). Our study consists with the previous research results. OR medical staffs who are ever exposed to COVID-19 patients are prone to experience psychological disorders. The high risk of occupational exposure might lead to psychological stress. Furthermore, the city lockdown and traffic restrictions limited the social activities of citizens, with associated increased depression and anxiety among the OR medical staffs, especially the medical staff living in suburb areas. In regard to the effect of city lockdown, 182 (92.4%) in the first survey considered their daily life were severely impacted by city lockdown. The high incidence of the anxiety and depression in OR medical staffs might be related to such factors as occupational exposure to novel coronavirus, a shortage of medical protective equipment, and lockdown of the city.

**Figure 2:**
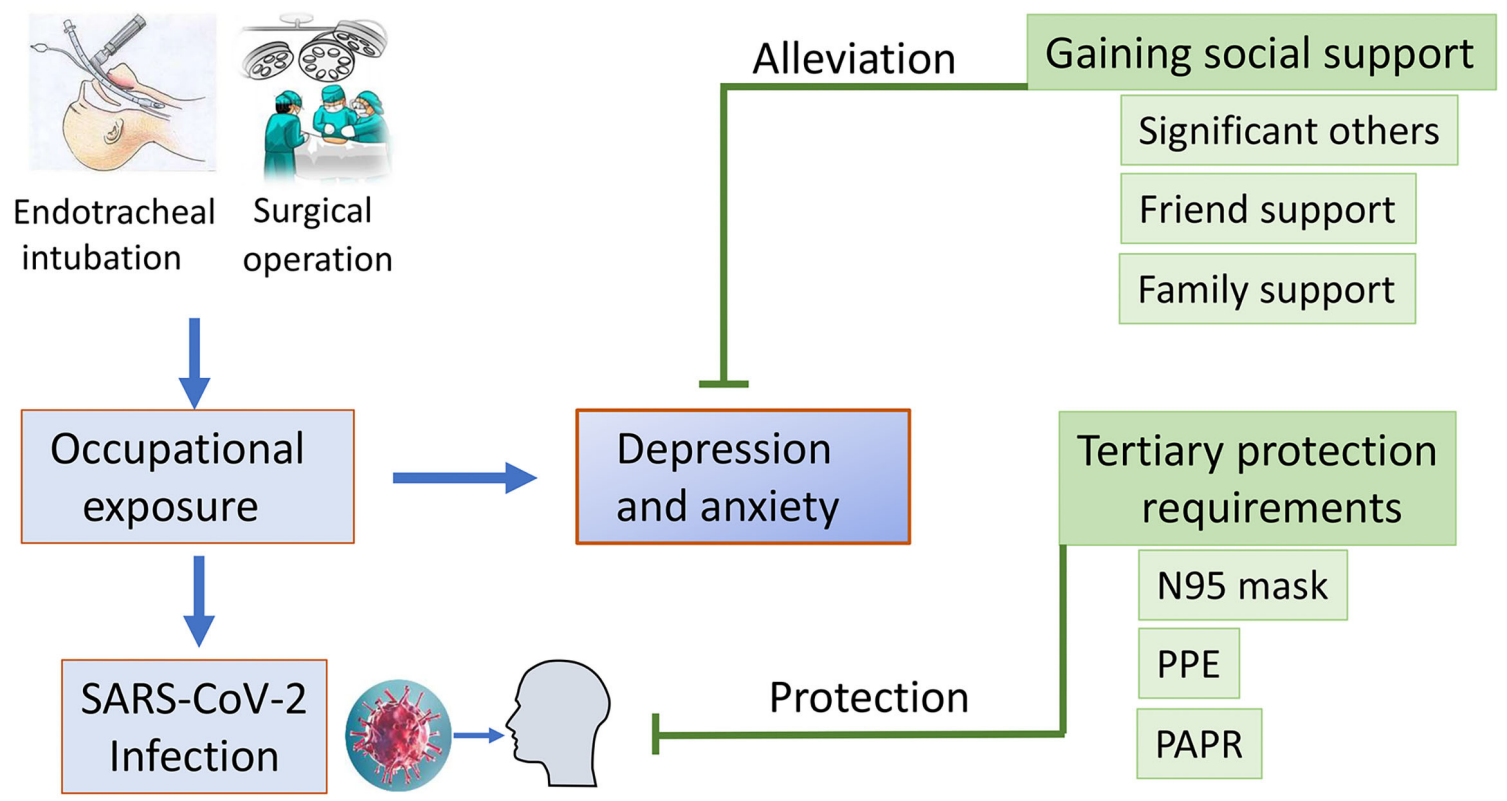
Infographic describing the association between occupational exposure, social support and development of depression and anxiety.

Our initial analysis and results prompted us to perform further data analysis. We also investigated perceived social support among the OR medical staffs. Our study showed that psychological distress in OR medical staffs was negatively correlated with perceived social support, especially friend support and significant other support. In this study, medical staffs in the secondary survey exhibited higher MSPSS scores than those in the first survey, suggesting that medical staffs gained more and more social support as better control of the COVID-19 epidemic has been achieved in Wuhan, China. This might be related to a series of encouragements from the media and the whole society, such as medical team reinforcements and the praise from the Chinese health authorities. Therefore, the medical staffs might alleviate the anxiety and depression accompanied by improved life quality with the help of international and national support. Several data have shown that perceived emotional and instrumental support from family, friends and perceived social support and recognition had significantly relieved anxiety (18, 26, 27). Consistently, our study revealed that social support was correlated with anxiety and depression in OR medical staffs, suggesting that the OR medical staffs might alleviate their depression by gaining social support (Figure 2). The present study highlights the importance of family support, friend support and social support, including the encouragements from family and friends, social recognition of the of medical staffs in this epidemic, praise from the media and the Chinese health authorities, which might be a potential strategy to alleviate the moderate psychological stress.

## Limitations

Our study has several limitations. Firstly, this was a single-center-based cohort study and all the participants were from Wuhan, which was limited in scope. Secondly, although our study suggested a high prevalence of psychological disorders after the outbreak of COVID-19 among the OR staffs, we had no data about the baseline depression/anxiety of the study participants before the SARS-COV-2 exposure. Besides, the first survey period was conducted during the “reducing stage” of COVID-19 outbreak, and the prevalence of depression and anxiety was possibly underestimated. Thirdly, this psychological assessment was based on an online survey and on self-report tools. We will use clinical interviews in future studies to draw a more comprehensive assessment of the problem.

## Conclusions

Due to the high risk of occupational exposure to COVID-19 and city lockdown, OR medical staffs exhibited high rates of depression and anxiety in this particular situation. Protecting OR medical staffs is an important component of public health measures for addressing the COVID-19 epidemic. OR medical staffs are in need of health protection including sufficient medical protective equipment, as well as special recovery programs aimed at improving psychological well-being. Furthermore, more social support and social recognition could be helpful for OR medical staffs to relieve their perceived psychological stress.

## Data Availability Statement

The raw data supporting the conclusions of this article will be made available by the authors, without undue reservation.

## Ethics Statement

The studies involving human participants were reviewed and approved by Ethics Committee of Zhongnan Hospital in Wuhan University. The ethics committee waived the requirement of written informed consent for participation.

## Author Contributions

X-yL designed the questionnaire and wrote the manuscript. JW collected the data. R-xZ, LC, and CH performed the data analysis. C-yW and X-mS darfted the form for Ethic Committee. Y-lW, Z-zZ, and J-jK designed the program and provided the professional guidance. All authors contributed to the article and approved the submitted version.

## Conflict of Interest

CH was employed by company Stego Tech LLC, King of Prussia, PA, USA, and worked the at department of Machine learning. The remaining authors declare that the research was conducted in the absence of any commercial or financial relationships that could be construed as a potential conflict of interest.

## Abbreviations

COVID-19: Coronavirus Disease 2019
SARS-Cov-2: SARS-Associated Coronavirus
OR: Operating Room
PHQ-9: Patient Health Questionaire-9
GAD-7: Generalized Anxiety Disorder-7
SF-36: The MOS Item Short From Health Survey
MSPSS: Multidimensional Scale of Perceived Social Support
PPE: personal protection equipment
PAPR: powered air-purifying respirator.
